# Theoretical Analysis of the Effects of Exothermic Catalytic Chemical Reaction on Transient Mixed Convection Flow along a Curved Shaped Surface

**DOI:** 10.3390/nano12244350

**Published:** 2022-12-07

**Authors:** Hossam A. Nabwey, Muhammad Ashraf, Uzma Ahmad, Ahmed. M. Rashad, Sumayyah I. Alshber, Miad Abu Hawsah

**Affiliations:** 1 Department of Mathematics, College of Science and Humanities in Al-Kharj, Prince Sattam bin Abdulaziz University, Al-Kharj 11942, Saudi Arabia; 2 Department of Basic Engineering Science, Faculty of Engineering, Menoufia University, Shebin El-Kom 32511, Egypt; 3 Department of Mathematics, Faculty of Science, University of Sargodha, Sargodha 40100, Pakistan; 4 Department of Mathematics, Faculty of Science, Aswan University, Aswan 81528, Egypt

**Keywords:** transient, exothermic catalytic chemical reaction, mixed convection, curved surface

## Abstract

The present problem addressed the transient behavior of convective heat and mass transfer characteristics across a curved surface under the influence of exothermic catalytic chemical reactions. The governing non-linear mathematical model wastransformed into a convenient form with the help of a primitive variable formulation. The final primitive formed model wassolved numerically by applying the finite difference method. The analysis of the above said computed numerical data in terms of oscillatory heat transfer, skin friction, and oscillatory mass transfer for various emerging parameters, such as the mixed convection parameter $\lambda_{T}$, modified mixed convection parameter $\lambda_{c}$, index parameter n, activation energy parameter E, exothermicparameter β, temperature relative parameter γ, chemical reaction parameter λ, and Schmidt number $S_{c}$ is plotted in graphical form. An excellent agreement is depicted for oscillatory heat transfer behavior at the large value of activation energy E. The amplitude of heat transfer and prominent fluctuating response in mass transfer with a certain height is found at each value of the index parameter n with a good alteration. An increase in the activation energy led to an increase in the surface temperature, which yielded more transient heat transfer in the above-said mechanism. The main novelty of the current study is that first, we ensured the numerical results for the steady state heat and fluid flow and then these obtained results wereused in the unsteady part to obtain numerical results for the transient behavior of the heat and mass transfer mechanism.

## 1. Introduction

The transient flow problems for steady and unsteady fluids with exothermic catalytic chemical reactions have various important applications in engineering and modern technologies, such as nuclear and thermal power plants, combustion chambers, thermal insulation of buildings, heat exchangers, and many more. In transient flows, the most important phenomenon is the species chemical reaction with finite Arrhenius activation energy. This is a very imperative factor that increases the temperature due to a marked increase in the reaction rates. Pop and Takhar [1] explored the free convection phenomenon along the curved surface to examine the heat transfer characteristics. Chamkha [2] numerically studied the unsteady laminar and electrical conducting fluid flow mechanism over a non-isothermal surface with an applied magnetic field under the influence of porous medium effects. He predicted that the wall heat transfer decreased due to strong magnetic field effects. Takhar et al. [3] developed the unsteady, magnetohydrodynamic, and heat transfer problem over a semi-infinite shape with free stream velocity and aligned magnetic field effects. The authors found that no change occurred in the velocity profile when the plate was moved in opposite directions to the free stream velocity. However, in the same direction as the free stream velocity, a change was noticed in the velocity profile. Chamkha [4] studied the transient and hydromagnetic fluid flow phenomena for heat transfer characteristics in circular pipes and channels by using a two-phase continuum model mathematically. He obtained the lower velocity gradients at the wall due to suspension in the channel or pipe, which was caused by strong magnetic field effects. Takhar et al. [5] considered the transient three-dimensional electrical conducting flow problem caused by the impulsive motion of the stretched shape along with surface temperature effects.

Magyari et al. [6] examined the computational behavior of a Darcy Boussinesq fluid along a permeable vertical shape immersed in the porous medium. They observed that the unique solution was obtained for the threshold value of the suction parameter $\gamma_{min},$ but multiple solutions were obtained for various values of γ. Later, Magyari et al. [7] critically observed the effects of free convective heat transfer along the curved surface. Chamkha et al. [8] obtained the numerical results of the oscillatory flow and heat transfer behavior in two immiscible fluids through the horizontal shape. They computed the oscillatory frequency and amplitude on the flow velocity and temperature field in tabular form. Later, Chamkha et al. [9] analytically analyzed the transient, laminar, and compressible boundary layer mechanism for hypersonic flow around a circular cone with suction effects near a plane of symmetry. Ishak et al. [10] examined the mixed convective heat transfer characteristics for the time-dependent flow of quiescent fluid over a stretchable vertical shape. Kabeir et al. [11] investigated the unsteady MHD mixed convective flow along a vertical moving shape immersed in a saturated porous medium by taking into account the uniform surface heat flux. They found that the heat transfer and skin friction were reduced at the stretching surface with a strong magnetic field but increased for the strong porous medium effects. Mahmood et al. [12] numerically investigated the thermal and mass diffusion effects on time-dependent mixed convection heat transfer phenomenon along a squeezing porous sensor shape placed inside a channel.

Maleque [13] discussed the MHD heat and mass transfer problem for Arrhenius activation energy with thermal radiation effects under the influence of exothermic/endothermic chemical reactions. Jha and Yousaf [14] analyzed the unsteady, viscous, and incompressible fully developed flow along a porous annular in the presence of isothermal heating effects. They depicted that the thermal transient was slower, and the conduction was less effective in the fluid due to the large value of the Prandtl number. Ashraf et al. [15] computed the viscous dissipation effects on thermal and periodic boundary layers of free-forced flow across the sphere. They obtained the strong time-dependent behavior of momentum boundary layer thickness at various positions of the sphere for different values of the Prandtl number Pr. Saha and Saha [16] analyzed the numerical solutions of the time-dependent mixed convection flow of incompressible fluid across the wedge with the effects of the applied magnetic field. They obtained the heat transfer and skin friction coefficients for various values of physical parameters numerically and graphically. Ashraf et al. [17] numerically explored the two-dimensional steady convective heat transfer flow along a curved shape under the influence of anexothermic catalytic chemical reaction. Later, Ahmad et al. [18] extended their work [17], including the effects of viscous dissipation. Ashraf and Ullah [19] computed the oscillating behavior of convective heat transfer in oscillatory fluid flow around a non-conducting circular shape in the presence of variable density effects. Ullah et al. [20,21,22] performed the magneto thermo analysis for fluctuating heat transfer behavior around various prominent positions of a non-conducting shape and under the influence of slip velocity, fluid viscosity, thermal conductivity, and thermal stratification effects. Ahmad et al. [23,24] investigated the mixed convection heat and mass transfer and the impact of variable viscosity and thermal conductivity on a chemically reacted curved surface. Further, Ashraf et al. [25,26,27] discussed the transient behavior of the chief physical quantities involved in heat and mass transfer mechanisms along different geometries.

Following [1] and [23], this work deals with the transient mixed convective incompressible flow of a two-dimensional viscous fluid along a curved shape. Equations for continuity, energy, momentum, and mass concentration are made dimensionless using appropriate dimensionless variables. Moreover, the transformed boundary layer equations are then simplified to algebraic equations using the finite difference method for the numerical investigation.

## 2. Mathematical Model and Solution Methodology

**Figure 1:**
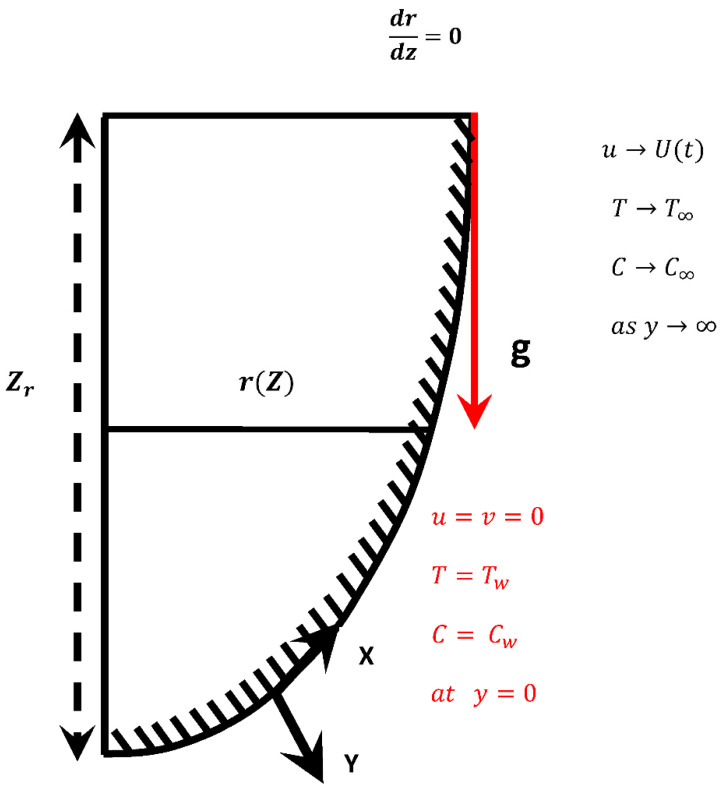
Coordinate system and flow configuration of the proposed model.

The proposed model highlights the phenomenon of transient, two-dimensional, viscous, and incompressible fluid flow in the presence of a catalytic chemical reaction. To calculate the transient behavior, we included the local acceleration, local temperature, and local mass concentration in the conservative equations (defined in [18]). The characteristics of the flow domain, along with the boundary condition, are represented in Figure 1. In this study, the distance x is measured along the curved surface and y is considered normal to it, where the effects of the solid and heated curved surface are examined.

When viscous dissipation is negligible, the conservative equations are reduced for the transient and incompressible fluid flow as given below:

(1) $$\frac{\partial \left(ud\right)}{\partial x}+\frac{\partial \left(vd\right)}{\partial y}=0,$$

(2) $$\frac{\partial u}{\partial t}+u\frac{\partial u}{\partial x}+v\frac{\partial u}{\partial y}=\nu \frac{\partial^{2}u}{\partial y^{2}}+g_{x}\beta_{T}\left(T-T_{\infty }\right)+g_{x}\beta_{C}\left(C-C_{\infty }\right),\;$$

(3) $$\frac{\partial T}{\partial t}+u\frac{\partial T}{\partial x}+v\frac{\partial T}{\partial y}=\alpha \frac{\partial^{2}T}{\partial y^{2}}+\beta k_{r}^{2}{\left(\frac{T}{T_{\infty }}\right)}^{n}e^{\frac{-E_{a}}{kT}}\left(T-T_{\infty }\right),$$

(4) $$\frac{\partial C}{\partial t}+u\frac{\partial C}{\partial x}+v\frac{\partial C}{\partial y}=D_{m}\frac{\partial^{2}C}{\partial y^{2}}+k_{r}^{2}{\left(\frac{T}{T_{\infty }}\right)}^{n}e^{\frac{-E_{a}}{kT}}\left(C-C_{\infty }\right)$$

with boundary conditions:

$$u=0,\;\;\;\;\;\;\;v=0,\;\;\;\;\;\;\;T=T_{w},\;\;\;\;\;\;\;C=C_{w}\;\;\;at\;\;\;y=0;$$

(5) $$u\to U\left(t\right),\;\;\;\;\;\;\;\;\;T\to T_{\infty },\;\;\;\;\;\;\;C\to C_{\infty }\;\;\;\;\;\;as\;\;\;\;\;\;\;y\to \infty .\;\;$$

In the above equations, $d=\sqrt{\frac{l}{\;g_{x}}}$, where $\;g_{x}$ is the x-component of acceleration due to gravity (defined in [17]). $\;g_{x}$ is added in the momentum conservative equation to shape the geometry as the curved surface. u and v are the *x* and *y*-components of velocity, $\beta_{T}$ is the coefficient of volumetric expansion due to temperature, $\beta_{C}$ is the coefficient of volumetric expansion due to mass concentration,  α is the thermal diffusivity, β is the exothermic parameter, $k_{r}^{2}$ is the chemical reaction rate constant, ${\left(\frac{T}{T_{\infty }}\right)}^{n}e^{\frac{-E_{a}}{kT}}$ is the Arrhenius function where n is the index ranging from −1 to 1, and $\;D_{m}$ is the mass diffusivity, respectively. The ambient fluid is assumed to be at a constant temperature $T_{\infty },$ and the reactant C at a constant concentration $C_{\infty }$. Moreover, U(t) is the free stream velocity.

### 2.1. Dimensionless Variables

The major advantage of non-dimensionalizing is the significant reduction in the number of parameters. The original problem involves ten parameters, but the non-dimensionalized problem is just based on six parameters. With this understanding, consider the following dimensionless variables:

x¯=xl,      y¯=ylRel12,       u¯=uUs,      v¯=vU∞Rel12,

(6) $$\theta =\frac{T-T_{\infty }}{T_{w}-T_{\infty }},\;\;\;\;\;\;\varphi =\frac{C-C_{\infty }}{C_{w}-C_{\infty }},\;\;\;\;\;\;\tau =\frac{U_{s}t}{l}$$

where the velocity scale and Reynolds number are defined as:

Us=(gxβ∆Tl)12,   Rel=Uslν

By using the above dimensionless variables that are given in (6), we obtainedthe dimensionless form (by dropping the bars) of the conservative Equations (1)–(4) along with the boundary conditions given in (5) as below;

(7) $$\frac{\partial u}{\partial x}+\frac{\partial v}{\partial y}=0$$

(8) $$\frac{\partial u}{\partial \tau }+u\frac{\partial u}{\partial x}+v\frac{\partial u}{\partial y}+\tilde{n}\frac{u^{2}}{2x}=\frac{\partial^{2}u}{\partial y^{2}}+\lambda_{T}\theta +\lambda_{C}\varphi ,$$

(9) $$\frac{\partial \theta }{\partial \tau }+u\frac{\partial \theta }{\partial x}+v\frac{\partial \theta }{\partial y}=\frac{1}{Pr}\frac{\partial^{2}\theta }{\partial y^{2}}+\beta \lambda^{2}\left(1+n\gamma \theta \right)\theta e^{\frac{-E}{1+\gamma \theta }},\;$$

(10) $$\frac{\partial \varphi }{\partial \tau }+u\frac{\partial \varphi }{\partial x}+v\frac{\partial \varphi }{\partial y}=\frac{1}{Sc}\frac{\partial^{2}\varphi }{\partial y^{2}}+\lambda^{2}\left(1+n\gamma \theta \right)\varphi e^{\frac{-E}{1+\gamma \theta }},$$

with the dimensionless boundary conditions:

u=0,    v=0,  θ=1,    φ=1     at        y=0

(11) u→U(τ),     θ→0,     ϕ→0       as    y→∞. 

In the above equations, $\lambda_{T}=\frac{Gr_{l}}{Re_{l}^{2,}}$ is the mixed convection parameter(also known as the Richardson parameter) and $\lambda_{C}=\frac{Gr_{l}^{*}}{Re_{l}^{2}}$ is the modified mixed convection parameter, where $Gr_{l}=\frac{g_{x}\beta_{T}∆Tl^{3}}{\nu^{2}},\;Gr_{l}{}^{*}=\frac{g_{x}\beta_{C}∆Cl^{3}}{\nu^{2}}.\;\;\lambda^{2}=\frac{k_{r}^{2}l}{U_{s}}$ is the dimensionless chemical reaction rate constant, and l is the characteristic length.$\gamma =\frac{T_{w}-T_{\infty }}{T_{\infty }}$ is the temperature relative parameter, $E=\frac{E_{a}}{kT_{\infty }}$ is the dimensionless activation energy, with $E_{a}$ as the activation energy, and $k=1.380649\times 10^{-23}JK^{-1}$ is the Boltzman constant. Moreover, Pr $=\frac{\nu }{\alpha }$ is the Prandtl number, and Sc $=\frac{\nu }{D_{m}}$ is the Schmidt number, respectively.

### 2.2. Stokes Conditions

Here, we define the free stream velocity $U\left(\tau \right)=1+\varepsilon e^{i\omega \tau }$ with |ε|<<1. Moreover, by using the oscillating Stokes condition, velocity components u and v, the temperature θ, and mass concentration φ can be written as the sum of steady and unsteady components given in Equation (12) (by following [15]).

(12) $$u=u_{s}+\varepsilon u_{t}e^{i\omega \tau },\;\;\;\;\;\;v=v_{s}+\varepsilon v_{t}e^{i\omega \tau },\;\;\;\;\;\;\;\;\varphi =\varphi_{s}+\varepsilon \varphi_{t}e^{i\omega \tau },\;\;\;\;\;\;\;\;\theta =\theta_{s}+\varepsilon \theta_{t}e^{i\omega \tau }$$

By using Equation (12) in thedimensionless Equations (7)–(10), we obtainedthe system of equations for non-oscillating and oscillating parts.

**Non-Oscillating Part**

(13) $$\frac{\partial u_{s}}{\partial x}+\frac{\partial v_{s}}{\partial y}=0,\;$$

(14) $$u_{s}\frac{\partial u_{s}}{\partial x}+v_{s}\frac{\partial u_{s}}{\partial y}+\frac{u_{s}^{2}}{2x}n=\frac{\partial^{2}u_{s}}{\partial y^{2}}+\lambda_{T}\theta_{s}+\lambda_{C}\varphi_{s},$$

(15) $$u_{s}\frac{\partial \theta_{s}}{\partial x}+v_{s}\frac{\partial \theta_{s}}{\partial y}=\frac{1}{Pr}\frac{\partial^{2}\theta_{s}}{\partial y^{2}}+\beta \lambda^{2}\left(1+n\gamma \theta_{s}\right)\left(1-E+E\gamma \theta_{s}\right)\theta_{s},$$

(16) $$u_{s}\frac{\partial \varphi_{s}}{\partial x}+v_{s}\frac{\partial \varphi_{s}}{\partial y}=\frac{1}{Sc}\frac{\partial^{2}\varphi_{s}}{\partial y^{2}}+\lambda^{2}\left(1+n\gamma \theta_{s}\right)\left(1-E+E\gamma \theta_{s}\right)\varphi_{s},$$

with the boundary conditions:

$$u_{s}=0,\;\;v_{s}=0,\;\;\theta_{s}=1,\;\;\varphi_{s}=1\;\;\;\;at\;y=0;$$

(17) $$u_{s}\to 1,\;\;\;\;\;\theta_{s}\to 0,\;\;\;\;\;\;\varphi_{s}\to 0\;\;\;\;as\;\;y\to \infty .$$

**Oscillating Part**

(18) $$\frac{\partial u_{t}}{\partial x}+\frac{\partial v_{t}}{\partial y}=0,$$

(19) $$i\omega u_{t}+u_{s}\frac{\partial u_{t}}{\partial x}+u_{t}\frac{\partial u_{s}}{\partial x}+v_{s}\frac{\partial u_{t}}{\partial y}+v_{t}\frac{\partial u_{s}}{\partial y}+\frac{u_{s}u_{t}}{x}n=\frac{\partial^{2}u_{t}}{\partial y^{2}}+\lambda_{T}\theta_{t}+\lambda_{C}\varphi_{t},$$

(20) $$\begin{array}{cc} i\omega \theta_{t}+u_{s}\frac{\partial \theta_{t}}{\partial x}+ & u_{t}\frac{\partial \theta_{s}}{\partial x}+v_{s}\frac{\partial \theta_{t}}{\partial y}+v_{t}\frac{\partial \theta_{s}}{\partial y}\\ & =\frac{1}{Pr}\frac{\partial^{2}\theta_{t}}{\partial y^{2}}+\beta \lambda^{2}\left[1+E\left(2\gamma \theta_{s}+3n\gamma^{2}\theta_{s}^{2}-1\right)\right]\theta_{t}, \end{array}$$

(21) $$\begin{array}{cc} i\omega \varphi_{t}+u_{s}\frac{\partial \varphi_{t}}{\partial x}+ & u_{t}\frac{\partial \varphi_{s}}{\partial x}+v_{s}\frac{\partial \varphi_{t}}{\partial y}+v_{t}\frac{\partial \varphi_{s}}{\partial y}\\ & =\frac{1}{Sc}\frac{\partial^{2}\varphi_{t}}{\partial y^{2}}\\ & +\lambda^{2}[\left(2E\gamma^{2}n\theta_{s}\varphi_{s}+E\gamma \varphi_{s}+n\gamma \varphi_{s}-nE\gamma \varphi_{s}\right)\theta_{t}\\ & +\left(1-E+n\gamma \theta_{s}-nE\gamma \theta_{s}+E\gamma \theta_{s}+nE\gamma^{2}\theta_{s}^{2}\right)\varphi_{t}],\; \end{array}$$

with the boundary conditions:

$$u_{t}=0,\;\;v_{t}=0,\;\;\theta_{t}=0,\;\;\varphi_{t}=0\;\;\;\;\;\;at\;y=0;$$

(22) $$u_{t}\to 1,\;\;\;\;\theta_{t}\to 0,\;\;\;\;\varphi_{t}\to 0\;\;\;\;\;\;as\;y\to \infty .$$

The oscillating part is further split up into the real and imaginary parts by considering the oscillating condition given in the following Stokes equations (by following [15]).

(23) $$u_{t}=u_{1}+iu_{2};\;\;\;\;v_{t}=v_{1}+iv_{2};\;\;\;\;\theta_{t}=\theta_{1}+i\theta_{2};\;\;\;\;\;\;\varphi_{t}=\varphi_{1}+i\varphi_{2},$$

Using Stokes Equation (23) in the unsteady model, Equations (18)–(21),and the boundary conditions (22), we have the real and imaginary parts as follows:

**Real Part**

(24) $$\frac{\partial u_{1}}{\partial x}+\frac{\partial v_{1}}{\partial y}=0,$$

(25) $$-\omega u_{2}+u_{s}\frac{\partial u_{1}}{\partial x}+u_{1}\frac{\partial u_{s}}{\partial x}+v_{s}\frac{\partial u_{1}}{\partial y}+v_{1}\frac{\partial u_{s}}{\partial y}+\frac{u_{s}u_{1}}{x}n=\frac{\partial^{2}u_{1}}{\partial y^{2}}+\lambda_{T}\theta_{1}+\lambda_{C}\varphi_{1},$$

(26) $$\begin{array}{cc} -\omega \theta_{2}+u_{s}\frac{\partial \theta_{1}}{\partial x} & +u_{1}\frac{\partial \theta_{s}}{\partial x}+v_{s}\frac{\partial \theta_{1}}{\partial y}+v_{1}\frac{\partial \theta_{s}}{\partial y}\\ & =\frac{1}{Pr}\frac{\partial^{2}\theta_{1}}{\partial y^{2}}+\beta \lambda^{2}\left[1+E\left(2\gamma \theta_{s}+3n\gamma^{2}\theta_{s}^{2}-1\right)\right]\theta_{1} \end{array}$$

(27) $$\begin{array}{cc} -\omega \varphi_{2}+u_{s}\frac{\partial \varphi_{1}}{\partial x} & +u_{1}\frac{\partial \varphi_{s}}{\partial x}+v_{s}\frac{\partial \varphi_{1}}{\partial y}+v_{1}\frac{\partial \varphi_{s}}{\partial y}\\ & =\frac{1}{Sc}\frac{\partial^{2}\varphi_{1}}{\partial y^{2}}\\ & +\lambda^{2}[\left(2E\gamma^{2}n\theta_{s}\varphi_{s}+E\gamma \varphi_{s}+n\gamma \varphi_{s}-nE\gamma \varphi_{s}\right)\theta_{1}\\ & +\left(1-E+n\gamma \theta_{s}-nE\gamma \theta_{s}+E\gamma \theta_{s}+nE\gamma^{2}\theta_{s}^{2}\right)\varphi_{1}],\; \end{array}$$

with the boundary conditions:

$$u_{1}=0,\;\;v_{1}=0,\;\;\theta_{1}=0,\;\;\varphi_{1}=0\;\;\;\;at\;\;\;\;y=0;$$

(28) $$u_{1}\to 1,\;\;\;\;\theta_{1}\to 0,\;\;\;\;\varphi_{1}\to 0\;\;\;\;\;\;as\;\;\;\;y\to \infty .$$

**Imaginary Part**

(29) $$\frac{\partial u_{2}}{\partial x}+\frac{\partial v_{2}}{\partial y}=0,$$

(30) $$\omega u_{1}+u_{s}\frac{\partial u_{2}}{\partial x}+u_{2}\frac{\partial u_{s}}{\partial x}+v_{s}\frac{\partial u_{2}}{\partial y}+v_{2}\frac{\partial u_{s}}{\partial y}+\frac{u_{s}u_{2}}{x}n=\frac{\partial^{2}u_{2}}{\partial y^{2}}+\lambda_{T}\theta_{2}+\lambda_{C}\varphi_{2},\;$$

(31) $$\begin{array}{cc} \omega \theta_{1}+u_{s}\frac{\partial \theta_{2}}{\partial x}+ & u_{2}\frac{\partial \theta_{s}}{\partial x}+v_{s}\frac{\partial \theta_{2}}{\partial y}+v_{2}\frac{\partial \theta_{s}}{\partial y}\\ & =\frac{1}{Pr}\frac{\partial^{2}\theta_{2}}{\partial y^{2}}+\beta \lambda^{2}\left[1+E\left(2\gamma \theta_{s}+3n\gamma^{2}\theta_{s}^{2}-1\right)\right]\theta_{2}, \end{array}$$

(32) $$\begin{array}{cc} \omega \varphi_{1}+u_{s}\frac{\partial \varphi_{2}}{\partial x}+ & u_{2}\frac{\partial \varphi_{s}}{\partial x}+v_{s}\frac{\partial \varphi_{2}}{\partial y}+v_{2}\frac{\partial \varphi_{s}}{\partial y}\\ & =\frac{1}{Sc}\frac{\partial^{2}\varphi_{2}}{\partial y^{2}}\\ & +\lambda^{2}[\left(2E\gamma^{2}n\theta_{s}\varphi_{s}+E\gamma \varphi_{s}+n\gamma \varphi_{s}-nE\gamma \varphi_{s}\right)\theta_{2}\\ & +\left(1-E+nE\gamma \theta_{s}-nE\gamma \theta_{s}+E\gamma \theta_{s}+nE\gamma^{2}\theta_{s}^{2}\right)\varphi_{2}], \end{array}$$

with boundary conditions

$$u_{2}=0,\;\;v_{2}=0,\;\;\theta_{2}=0,\;\;\varphi_{2}=0\;\;\;\;at\;\;\;y=0;$$

(33) $$u_{2}\to 0,\;\;\;\;\;\;\;\theta_{2}\to 0,\;\;\;\;\;\;\;\varphi_{2}\to 0\;\;\;\;\;\;as\;\;\;y\to \infty .$$

### 2.3. Primitive Variable Formulation

The primitive forms of the non-oscillating and oscillating models are given below by applying the given primitive variables for each model (following [21]).

**For Non oscillating Part**

us=Us(X,Y),  vs=x−12Vs(X,Y),         x=X,  y=x12Y,  

(34) $$\theta_{s}=\Theta_{s}\left(X,Y\right),\;\;\;\;\;\;\;\;\varphi_{s}=\Phi_{s}\left(X,Y\right),$$

By using Equation (34) into Equations (13)–(16) and the boundary conditions (17), we have:

(35) $$X\frac{\partial U_{s}}{\partial X}-\frac{Y}{2}\frac{\partial U_{s}}{\partial Y}+\frac{\partial V_{s}}{\partial Y}=0,\;$$

(36) $$\frac{n}{2}U_{s}^{2}+XU_{s}\frac{\partial U_{s}}{\partial X}+\left(V_{s}-\frac{YU_{s}}{2}\right)\frac{\partial U_{s}}{\partial Y}=\frac{\partial^{2}U_{s}}{\partial Y^{2}}+\lambda_{T}\Theta_{s}+\lambda_{C}\Phi_{s},$$

(37) $$XU_{s}\frac{\partial \Theta_{s}}{\partial X}+\left(V_{s}-\frac{YU_{s}}{2}\right)\frac{\partial \Theta_{s}}{\partial Y}=\frac{1}{Pr}\frac{\partial^{2}\Theta_{s}}{\partial Y^{2}}+\beta \lambda^{2}\left(1+n\gamma \Theta_{s}\right)\left(1-E+E\gamma \Theta_{s}\right)\Theta_{s},$$

(38) $$XU_{s}\frac{\partial \Phi_{s}}{\partial X}+\left(V_{s}-\frac{YU_{s}}{2}\right)\frac{\partial \Phi_{s}}{\partial Y}=\frac{1}{Sc}\frac{\partial^{2}\Phi_{s}}{\partial Y^{2}}+\lambda^{2}\left(1+n\gamma \Theta_{s}\right)\left(1-E+E\gamma \Theta_{s}\right)\Phi_{s}.$$

The transformed boundary conditions are:

$$U_{s}=0,\;\;\;\;\;\;\;\;\;V_{s}=0,\;\;\;\;\;\;\;\;\Theta_{s}=1,\;\;\Phi_{s}=1\;\;\;\;\;\;\;at\;\;\;\;\;\;\;\;\;Y=0$$

(39) $$U_{s}\to 1,\;\;\;\;\Theta_{s}\to 0,\;\;\;\Phi_{s}\to 0\;\;\;\;\;as\;\;\;Y\to \infty$$

**For Real Part**

u1=U1(X,Y),  v1=x−12V1(X,Y),         x=X,  y=x12Y,  

(40) $$\theta_{1}=\Theta_{1}\left(X,Y\right),\;\;\;\;\;\;\;\;\varphi_{1}=\Phi_{1}\left(X,Y\right),$$

By using Equation (40) into Equations (24)–(27) and the boundary conditions (28), we have:

(41) $$X\frac{\partial U_{1}}{\partial X}-\frac{Y}{2}\frac{\partial U_{1}}{\partial Y}+\frac{\partial V_{1}}{\partial Y}=0,\;$$

(42) $$\begin{array}{cc} -\omega XU_{2+}X(U_{s} & \frac{\partial U_{1}}{\partial X}+U_{1}\frac{\partial U_{s}}{\partial X})+\left(V_{s}-\frac{YU_{s}}{2}\right)\frac{\partial U_{1}}{\partial Y}+\left(V_{1}-\frac{YU_{1}}{2}\right)\frac{\partial U_{s}}{\partial Y}\\ & +U_{1}U_{s}n=\frac{\partial^{2}U_{1}}{\partial Y^{2}}+\lambda_{T}\Theta_{1}+\lambda_{C}\Phi_{1}, \end{array}$$

(43) $$\begin{array}{cc} -\omega X\Theta_{2}+X(U_{s} & \frac{\partial \Theta_{1}}{\partial X}+U_{1}\frac{\partial \Theta_{s}}{\partial X})+\left(V_{s}-\frac{YU_{s}}{2}\right)\frac{\partial \Theta_{1}}{\partial Y}+\left(V_{1}-\frac{YU_{1}}{2}\right)\frac{\partial \Theta_{s}}{\partial Y}\\ & =\frac{1}{Pr}\frac{\partial^{2}\Theta_{1}}{\partial Y^{2}}+\beta \lambda^{2}X\left[\left(1+E(2\gamma \Theta_{s}+3n\gamma^{2}\Theta_{s}^{2}-1\right)\right]\Theta_{1}, \end{array}$$

(44) $$\begin{array}{cc} -\omega X\Phi_{2}+X(U_{s} & \frac{\partial \Phi_{1}}{\partial X}+U_{1}\frac{\partial \Phi_{s}}{\partial X})+\left(V_{s}-\frac{YU_{s}}{2}\right)\frac{\partial \Phi_{1}}{\partial Y}+\left(V_{1}-\frac{YU_{1}}{2}\right)\frac{\partial \Phi_{s}}{\partial Y}\\ & =\frac{1}{Sc}\frac{\partial^{2}\Phi_{1}}{\partial Y^{2}}+\lambda^{2}X[(2E\gamma^{2}+n\Theta_{s}\Phi_{s}+E\gamma \Phi_{s}+n\gamma \Phi_{s}\\ & -n\gamma E\Phi_{s})\Theta_{1}(1-E+nE\gamma \Theta_{s}-nE\gamma \Theta_{s}+E\gamma \Theta_{s}\\ & +nE\gamma^{2}\Theta_{s}^{2})\Phi_{1}] \end{array}$$

The transformed boundary conditions are:

$$U_{1}=0,\;\;\;\;\;\;\;\;\;V_{1}=0,\;\;\;\;\;\;\;\;\Theta_{1}=0,\;\;\Phi_{1}=0\;\;\;\;\;\;\;at\;\;\;\;\;\;\;\;\;Y=0$$

(45) $$U_{1}\to 1,\;\;\;\;\Theta_{1}\to 0,\;\;\;\Phi_{1}\to 0\;\;\;\;\;as\;\;\;Y\to \infty$$

**For Imaginary Part:**

u2=U2(X,Y),  v2=x−12V2(X,Y),         x=X,  y=x12Y,  

(46) $$\theta_{2}=\Theta_{2}\left(X,Y\right),\;\;\;\;\;\;\;\;\varphi_{2}=\Phi_{2}\left(X,Y\right),$$

By using Equation (46) into Equations (29)–(32) along with the boundary conditions (33), we have:

(47) $$X\frac{\partial U_{2}}{\partial X}-\frac{Y}{2}\frac{\partial U_{2}}{\partial Y}+\frac{\partial V_{2}}{\partial Y}=0,\;$$

(48) $$\begin{array}{cc} -\omega XU_{1}+X(U_{s} & \frac{\partial U_{2}}{\partial X}+U_{2}\frac{\partial U_{s}}{\partial X})+\left(V_{s}-\frac{YU_{s}}{2}\right)\frac{\partial U_{2}}{\partial Y}+\left(V_{2}-\frac{YU_{2}}{2}\right)\frac{\partial U_{s}}{\partial Y}\\ & +U_{2}U_{s}n=\frac{\partial^{2}U_{2}}{\partial Y^{2}}+\lambda_{T}\Theta_{2}+\lambda_{C}\Phi_{2} \end{array}$$

(49) $$\begin{array}{cc} -\omega X\Theta_{1}+X(U_{s} & \frac{\partial \Theta_{2}}{\partial X}+U_{2}\frac{\partial \Theta_{s}}{\partial X})+\left(V_{s}-\frac{YU_{s}}{2}\right)\frac{\partial \Theta_{2}}{\partial Y}+\left(V_{2}-\frac{YU_{2}}{2}\right)\frac{\partial \Theta_{s}}{\partial Y}\\ & =\frac{1}{Pr}\frac{\partial^{2}\Theta_{2}}{\partial Y^{2}}+\beta \lambda^{2}X\left[\left(1+E(2\gamma \Theta_{s}+3n\gamma^{2}\Theta_{s}^{2}-1\right)\right]\Theta_{2} \end{array}$$

(50) $$\begin{array}{cc} -\omega X\Phi_{1}+X(U_{s} & \frac{\partial \Phi_{2}}{\partial X}+U_{2}\frac{\partial \Phi_{s}}{\partial X})+\left(V_{s}-\frac{YU_{s}}{2}\right)\frac{\partial \Phi_{2}}{\partial Y}+\left(V_{1}-\frac{YU_{2}}{2}\right)\frac{\partial \Phi_{s}}{\partial Y}\\ & =\frac{1}{Sc}\frac{\partial^{2}\Phi_{2}}{\partial Y^{2}}+\lambda^{2}X[(2E\gamma^{2}+n\Theta_{s}\Phi_{s}+E\gamma \Phi_{s}+n\gamma \Phi_{s}\\ & -n\gamma E\Phi_{s})\Theta_{2}(1-E+nE\gamma \Theta_{s}-nE\gamma \Theta_{s}+E\gamma \Theta_{s}\\ & +nE\gamma^{2}\Theta_{s}^{2})\Phi_{2}] \end{array}$$

The transformed boundary conditions are:

$$U_{2}=0,\;\;\;\;\;\;\;\;\;V_{2}=0,\;\;\;\;\;\;\;\;\Theta_{2}=0,\;\;\Phi_{2}=0\;\;\;\;\;\;\;at\;\;\;\;\;\;\;\;\;Y=0$$

(51) $$U_{2}\to 0,\;\;\;\;\Theta_{2}\to 0,\;\;\;\Phi_{2}\to 0\;\;\;\;\;as\;\;\;Y\to \infty$$

The FDM method is used to solve the previously mentioned primitive generated Equations (34)–(51) with the Gaussian elimination technique (as provided in [21]) for skin friction $\tau_{skin}$, heat transfer $\tau_{heat},$ and mass transfer $\tau_{mass}$ at various positions along the curved surface.

$$\tau_{skin}={\left(\frac{\partial U}{\partial Y}\right)}_{y=0}+\varepsilon \left|A_{s}\right|cos\left(\omega t+\alpha_{s}\right),\;\;\;\;\;$$

(52) $$\tau_{heat}={\left(\frac{\partial \theta }{\partial Y}\right)}_{y=0}+\varepsilon \left|A_{t}\right|cos\left(\omega t+\alpha_{t}\right),\;\;\;$$

$$\tau_{mass}={\left(\frac{\partial \Phi }{\partial Y}\right)}_{y=0}+\varepsilon \left|A_{m}\right|cos\left(\omega t+\alpha_{m}\right),$$

where $A_{s},A_{t}$, and $A_{m}$ are the amplitudes and $\alpha_{s}$, $\alpha_{t}$, and $\alpha_{m}$ are the phase angles defined as:

$$A_{s}={\left(u_{1}{}^{2}+u_{2}{}^{2}\right)}^{\frac{1}{2}},\;\;\;\;\;\;\;\;\;A_{t}={\left(\theta_{1}{}^{2}+\theta_{2}{}^{2}\right)}^{\frac{1}{2}},\;\;\;\;\;\;\;\;A_{m}={\left(\Phi_{x1}{}^{2}+\Phi_{x2}{}^{2}\right)}^{\frac{1}{2}},$$

αs=tan−1(u2u1),       αt=tan−1(θ2θ1),     αm=tan−1(φx2φx1).

## 3. Results and Discussion

In the present section, we consider in further detail the transient mixed convection flow along a curved surface with exothermic catalytic chemical reactive effects. Because the flow is unsteady throughout, the amplitude and phase angle can be involved andfurther used to calculate the transient behavior of the above said mathematical model given in Equations (18)–(21) along with boundary conditions (22). To overcome the non-linearity involved in the model, the numerical value of the steady terms and their derivatives can be used from the steady part.

**Figure 2:**
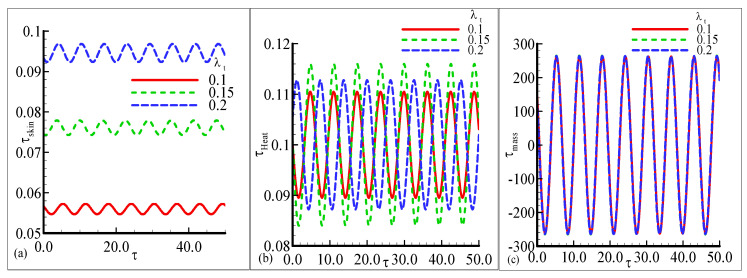
The physical profiles for $\tau_{skin}$, $\tau_{heat},$ and $\tau_{mass}$ with choices of $\lambda_{t}=0.1,\;0.15,$ and 0.2 and fixed Pr =7.0, $S_{c}=0.2$, $\lambda_{c}=0.6$, E=0.1, γ=0.1, λ=0.2, β=0.5, n=0.3, and ϵ=0.05.

**Figure 3:**
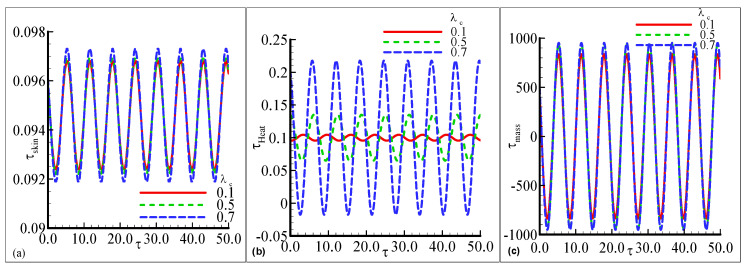
The physical profiles for $\tau_{skin}$, $\tau_{heat,}$ and $\tau_{mass}$ with choices of $\lambda_{c}=0.1,\;0.5,$ and 0.7 and fixed Pr =7.0, $S_{c}=0.2$, $\lambda_{t}=0.5$, E=0.1, γ=0.3, λ=0.5, β=0.2, n=0.3, and ϵ=0.05.

Figure 2a–c illustrates the oscillating behavior of heat transfer, skin friction, and mass transfer along a curved shape for parameter $\lambda_{t}=0.1,\;0.15,$ and 0.2 while other parameters are fixed. The value of skin friction is maximum at $\lambda_{t}=0.2$ with a small amplitude and is minimum at a lower value of $\lambda_{t}=0.1$ in Figure 2a. In Figure 2b, the maximum oscillation in heat transfer is noted for each value of $\lambda_{t}$. The heat transfer is maximum at $\lambda_{t}=0.15$ with a prominent amplitude. The oscillatory mass transfer is uniformly distributed for each value of $\lambda_{t}$ with the maximum amplitude in Figure 2c. The fluctuating behavior of $\tau_{heat}$, $\tau_{skin},\;$and oscillatory $\tau_{mass}$ is concluded for different values of the modified Richardson parameter $\lambda_{c}=0.1,\;0.5$ and 0.7 in Figure 3a–c. The uniform oscillating response of $\tau_{skin}$, and $\tau_{mass}$ is noted at each value of $\lambda_{c}$ but small change is observed for the large value of $\lambda_{c}=0.7$ with a high amplitude in Figure 3a,c. The prominent amplitude of oscillation in $\tau_{heat}$ is noted for different values of $\lambda_{c}$ but the highest oscillation is observed for a large value of $\lambda_{c}=0.7$ in Figure 3b. The small wave-like behavior is depicted in oscillatory heat transfer at lower $\lambda_{c}=0.1$. This above said mechanism is expected because, due to the mixed convection, the buoyancy forces act similar topressure gradients which yield an increase in the amplitude ofeach profile.

**Figure 4:**
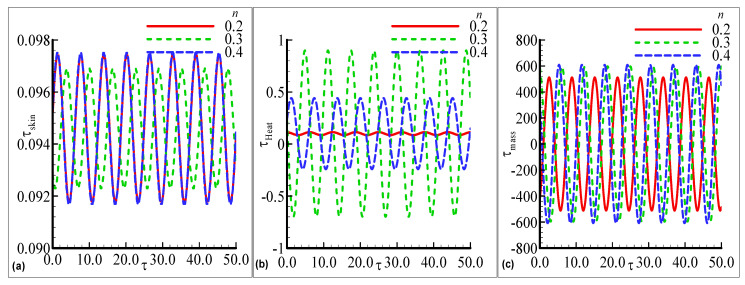
The physical profiles for $\tau_{skin}$, $\tau_{heat},$ and $\tau_{mass}$ with choices of n=0.2, 0.3, and 0.4 and fixed Pr =7.0, $S_{c}=0.2$, $\lambda_{t}=0.7$, $\lambda_{c}=0.6,E=0.1$, γ=0.3, λ=0.5, β=0.2 and ϵ=0.05.

**Figure 5:**
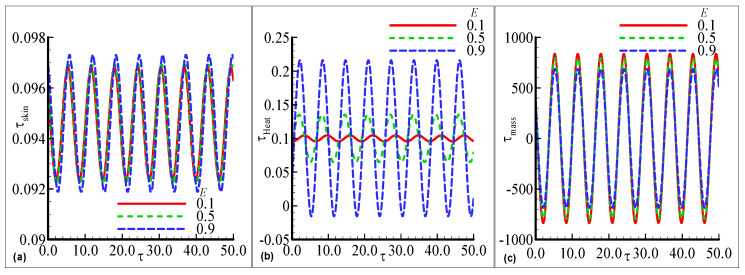
The physical profiles for $\tau_{skin}$, $\tau_{heat},$ and $\tau_{mass}$ with choices of E=0.1, 0.5, and 0.9 and fixed Pr =7.0, $S_{c}=0.2$, $\lambda_{t}=0.2$, $\lambda_{c}=0.6,\;n=0.3$, γ=0.3, λ=0.2, β=0.2, and ϵ=0.05.

**Figure 6:**
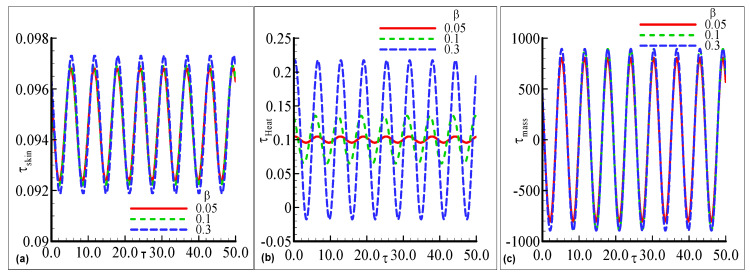
The physical profiles for $\tau_{skin}$, $\tau_{heat}$, and $\tau_{mass}$ with choices of β=0.05, 0.1, and 0.3 and fixed Pr =7.0, $S_{c}=0.2$, $\lambda_{t}=0.4$, $\lambda_{c}=0.6,E=0.7$, γ=0.3, λ=0.2, n=0.3, and ϵ=0.05.

Figure 4a–c demonstrates the maximum fluctuations in each profile for every value of the index parameter n=0.2, 0.3, and 0.4 with the prominent distribution. The maximum amplitude is observed for two choices of n=0.2 and 0.4 uniformly but small oscillations in the skin friction arenoted at n=0.3 in Figure 4a. In Figure 4b, the large amplitude in the heat transfer with a certain height is examined at n=0.3 but a small oscillation is computed at lower n=0.2. In Figure 4c, the prominent fluctuating response in mass transfer with good variations at each value of the index parameter n is noted but is maximum for the largest value n=0.4. The oscillating $\tau_{skin}$, $\tau_{heat,}$ and $\tau_{mass}$ for three choices of the activation energy parameter E=0.1, 0.5 and 0.9 are displayed in Figure 5a–c. The oscillatory skin friction and oscillatory mass transfer are uniformly distributed for each value of E. The value of heat transfer is maximum at the largest E=0.9 but the minimum value is observed for the smallest E=0.1. Since, an increase in activation energy leads to an increase in surface temperature, ityields more heat transfer in the above-said phenomena. The effects of the exothermic parameter β on oscillatory $\tau_{skin}$, $\tau_{heat,}$ and $\tau_{mass}$ profiles are plotted in Figure 6a–c. $\tau_{skin}$, and $\tau_{mass}$ are uniformly distributed at each value of β but the maximum amplitude is depicted for the large value of β=0.3 in Figure 6a,c. The highest oscillations are depicted in the heat transfer with prominent change, but it is maximum at a higher value of β=0.3 in Figure 6b. The small fluctuating behavior in heat transfer is concluded at the lowest value of β=0.05.

**Figure 7:**
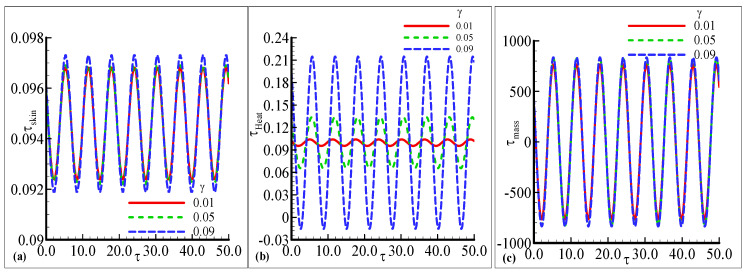
The physical profiles for $\tau_{skin}$, $\tau_{heat,}$ and $\tau_{mass}$ with choices of γ=0.01, 0.05, and 0.09 and fixed Pr =7.0, $S_{c}=0.2$, $\lambda_{t}=0.2$, $\lambda_{c}=0.6,E=0.2$, n=0.3, λ=0.2, β=0.2, and ϵ=0.05.

**Figure 8:**
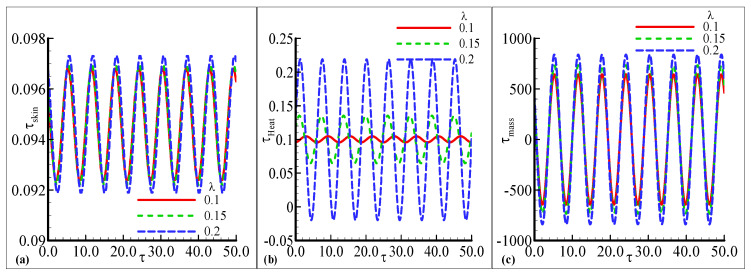
The physical profiles for $\tau_{skin}$, $\tau_{heat},$ and $\tau_{mass}$ with choices of λ=0.1, 0.15, and 0.2 and fixed Pr =7.0, $S_{c}=0.2$, $\lambda_{t}=0.5$, $\lambda_{c}=0.6,E=0.2$, γ=0.3, n=0.3, β=0.5, and ϵ=0.05.

**Figure 9:**
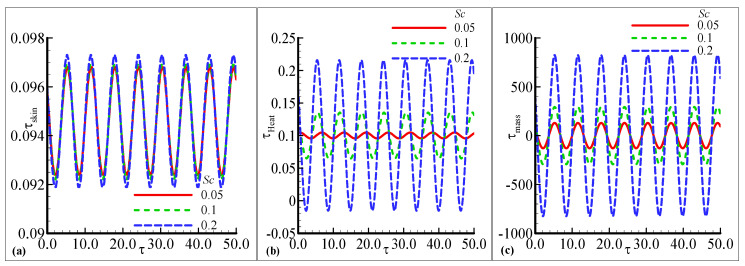
The physical profiles for $\tau_{skin}$, $\tau_{heat,}$ and $\tau_{mass}$ with choices of $S_{c}=0.05,\;0.1,$ and 0.2 and fixed Pr =7.0, λ=0.2, $\lambda_{t}=0.5$, $\lambda_{c}=0.6,E=0.2$, γ=0.3, n=0.3, β=0.5, and ϵ=0.05.

The amplitude of oscillation in $\tau_{skin}$, $\tau_{heat,}$ and $\tau_{mass}$ under the influence of the temperature-relative parameter γ are plotted against some fixed parameters in Figure 7a–c. The oscillating skin friction and mass transfer exhibit similar behavior for three choices of γ=0.01, 0.05, and 0.09 but a small change is noted at a certain height in Figure 7a,c. The amplitude is increased as the value of γ is increased. The prominent change in oscillations of $\tau_{heat}$ is observed for every value of γ in Figure 7b. The oscillatory heat transfer is increased with the highest amplitude γ is increased, but a very small oscillating response is noted at γ=0.01. Figure 8a,c demonstrate the oscillating response of skin friction and mass transfer with small changes against three choices of the chemical reaction parameter λ=0.1, 0.15 and 0.2. The uniform oscillation in both skin friction and mass transfer is noted with small variation for each choice of λ. The maximum amplitude is examined with a certain height as the value of λ is increased. The favorable transient response in $\tau_{heat}$ is noted and also the maximum amplitude is observed as λ is increased in Figure 8b. The minimum amplitude response is depicted as λ is decreased. The fluctuating behavior of heat transfer with good variations for each value of λ is illustrated in Figure 8b. Figure 9b,c illustrates the good oscillating response in the heat and mass transfer with prominent variations at each choice of the Schmidt number $S_{c}=0.05,\;0.1,$ and 0.2 with some fixed variations. The maximum amplitude of oscillation in the heat and mass transfer is at a large value of $S_{c}=0.05$ but the oscillating mass transfer is more favorable than oscillatory heat transfer. It is also depicted that the most prominent change in the mass transfer is noted for $S_{c}$ than the other physical parameters. The uniform response in skin friction is noted with the high amplitude at $S_{c}=0.2$ in Figure 9a. Asmall change is noticed at the peak amplitude of skin friction. As a result and with reference the comparison of the obtained results for the rate of heat transfer by the present author and Pop et al. [1] is given in Table 1 for different values of the index parameter *n* and Pr = 1.0 which support the main findings given in the current study.

## 4. Conclusions

The transient behavior of the mixed convective heat and mass transfer characteristics over a curved surface and under the influence of an exothermic catalytic chemical reaction has been addressed. Numerical simulations have been conducted in order to examine the effects of different parameters involved in the flow model. The main findings are summarized in the following paragraphs.

- It is seen that the transient rate of skin friction has improved for $\lambda_{T}=0.2$. On the other hand, the transient rate of heat transfer increased with a prominent amplitude for $\lambda_{T}=0.15$.
- A uniform behavior of the transient rate of skin friction and mass transfer is noted for different values of $\lambda_{C}$, while a prominent amplitude of oscillation in the case of the transient rate of heat transfer has been observed.
- A very strong role of body shape n was observed in terms of transient skin friction, and mass transfer.
- The transient rate of skin friction and mass transfer are uniform in terms of E, but the transient rate of heat transfer is increased for higher values of the dimensionless activation energy parameter E.
- It is noted that the amplitude of oscillation in terms of the transient skin friction and mass transfer is uniform and is maximum for the highest value of β, but on the other hand, the amplitude of oscillation for heat transfer is very small, similar to aslow pulse.
- The prominent change for every value of dimensionless temperature relative parameter γ in the case of the transient heat transfer has been depicted.
- The prominent oscillatory response in the heat transfer for different values of λ has been observed, and the smallest amplitude for the lowest value of λ has been noted.
- A significant illustration in the transient heat and mass transfer for prominent variation in the Schmidt number Sc has been noted.
- The current study can be extended for the entropy analysis of the transient mixed convection flow along the curved surface and in the presence of an exothermic catalytic chemical reaction.

## Figures and Tables

**Table 1:** Comparison of the obtained results for rate of heat transfer by the present author and Pop et al. [1] for different values of index parameter *n* and Pr = 1.0.

*n*	Present	Pop and Takhar [1]
0.1	0.3618	0.3690
0.2	0.3457	0.3469
0.3	0.2944	0.2949
0.4	0.24107	0.2488
0.5	0.19203	0.1946

## Data Availability

Data are available upon request.

## Nomenclature

x	Axis along the curved surface (m)
y	Axis normal to the curved surface (m)
u	Velocity along x-axis (m/s)
v	Velocity along y-axis (m/s)
d	(m)
t	Time (s)
T	Dimensioned temperature (K)
$T_{\infty }$	Ambient fluid temperature (K)
$T_{w}$	Temperature of the surface (K)
C	Dimensioned mass concentration (Kg/$m^{3}$)
$C_{w}$	Mass concentration at the surface (Kg/$m^{3}$)
$C_{\infty }$	Ambient mass concentration (Kg/$m^{3}$)
$g_{x}$	Tangential component of acceleration due to gravity $\left(m/s^{2}\right)$
$Re_{l}$	Local Reynolds number
$Gr_{x}$	Grashoff number
$Gr_{x}^{*}$	Modified Grashoff number
Sc	Schmidth number
Pr	Prandtl number
k	Boltzman constant
$k_{r}^{2}$	Chemical reaction rate constant
n	Index parameter lies in range
$E_{a}$	Activation energy
E	Dimensionless activation energy
U(t)	Free stream velocity (m/s)
l	Characteristic Length (m)
$D_{m}$	Mass diffusivity coefficient
**Greek letters**	
θ	Dimensionless temperature
φ	Dimensionless mass concentration
β	Exothermic parameter
$\beta_{T}$	Coefficient of volumetric expansion due to temperature
$\beta_{c}$	Coefficient of volumetric expansion due to mass concentration
α	Thermal diffusivity coefficient $\left(m^{2}/s\right)$
γ	Temperature relative parameter
τ	Dimensionless time
λ	Dimensionless chemical reaction rate constant
$\lambda_{T}$	Mixed convection parameter
$\lambda_{C}$	Modified mixed convection parameter
ε	Amplitude of oscillation (m)
ω	Frequency parameter (Hz)
κ	Thermal conductance of fluid (m-K)
σ	Electrical conductivity (Siemens/meter (s/m))
